# Impact of the Trifecta bioprosthetic valve in patients with low-flow severe aortic stenosis

**DOI:** 10.1007/s00380-021-01802-5

**Published:** 2021-02-14

**Authors:** Tohru Takaseya, Atsunobu Oryoji, Kazuyoshi Takagi, Tomofumi Fukuda, Koichi Arinaga, Shinichi Hiromatsu, Eiki Tayama

**Affiliations:** Department of Surgery, Kurume University, Asahi-machi 67, Kurume-shi, Fukuoka Japan

**Keywords:** Aortic valve replacement, Trifecta bioprosthesis, Left ventricular dysfunction, Patient-prosthesis mismatch

## Abstract

Aortic stenosis (AS) is the most common valve disorder in advanced age. Previous reports have shown that low-flow status of the left ventricle is an independent predictor of cardiovascular mortality after surgery. The Trifecta bioprosthesis has recently shown favorable hemodynamic performance. This study aimed to evaluate the effect of the Trifecta bioprosthesis, which has a large effective orifice area, in patients with low-flow severe AS who have a poor prognosis. We retrospectively evaluated 94 consecutive patients with severe AS who underwent aortic valve replacement (AVR). Patients were divided into two groups according to the stroke volume index (SVI): low-flow (LF) group (SVI < 35 ml/m^2^, *n* = 22) and normal-flow (NF) group (SVI ≥ 35 ml/m^2^, *n* = 72). Patients’ characteristics and early and mid-term results were compared between the two groups. There were no differences in patients’ characteristics, except for systolic blood pressure (LF:NF = 120:138 mmHg, *p* < 0.01) and the rate of atrial fibrillation between the groups. A preoperative echocardiogram showed that the pressure gradient was higher in the NF group than in the LF group, but aortic valve area was similar. The Trifecta bioprosthesis size was similar in both groups. The operative outcomes were not different between the groups. Severe patient–prosthesis mismatch (PPM) (< 0.65 cm^2^/m^2^) was not observed in either of the groups. There were no significant differences in mid-term results between the two groups. The favorable hemodynamic performance of the Trifecta bioprosthesis appears to have the similar outcomes in the LF and NF groups. AVR with the Trifecta bioprosthesis should be considered for avoidance of PPM, particularly in AS patients with LV dysfunction.

## Introduction

Aortic stenosis (AS) is the most common valve disorder and the most frequent indication for surgical treatment, aortic valve replacement (AVR), or transcatheter aortic valve implantation (TAVI) in advanced age. Previous reports have shown that low-flow status of the left ventricle (LV) is an independent predictor of cardiovascular mortality and long-term results after AVR or TAVI [1–6]. However, evaluating the low-flow status in severe AS patients is difficult because the reasons for low flow are multifactorial, including impaired myocardial contractility, restrictive physiological features, and afterload mismatch with high valvuloarterial impedance [7]. An example of this situation is that the LV ejection fraction does not adequately reflect total LV function in a setting of marked LV hypertrophy and relatively small LV volumes typical of high-grade AS. Recently, the stroke volume index (SVI), which is measured in LV outflow in pulsed-wave Doppler recordings, has been considered the most comprehensive indicator of LV function. Previous reports [1–6] have shown that a low SVI is an independent predictor of operative or long-term outcomes.

Another potential problem in AVR using a bioprosthesis for treating severe AS with LV dysfunction is a high incidence of patient–prosthesis mismatch (PPM). Blais et al. reported that PPM is associated with increased operative mortality after AVR, particularly when associated with LV dysfunction [8]. The Trifecta bioprosthesis (Abbott, Minneapolis, MN, USA) is a tri-leaflet, stented, bovine pericardial valve that was designed for supra-annular placement in the aortic position. The bovine pericardial sheet is mounted outside the stent frame, which allows for almost circular cross section during systole. Several reports have shown a favorable hemodynamic profile for this bioprosthesis, such as low peak and mean trans-prosthetic gradients, an excellent effective orifice area (EOA), and a low incidence of PPM in patients with a small aortic annulus [9, 10]. This study aimed to evaluate the effect of the Trifecta bioprosthesis, which has a large effective orifice area, in patients with low-flow AS who have a poor prognosis.

## Materials and methods

### Study population

**Fig. 1:**
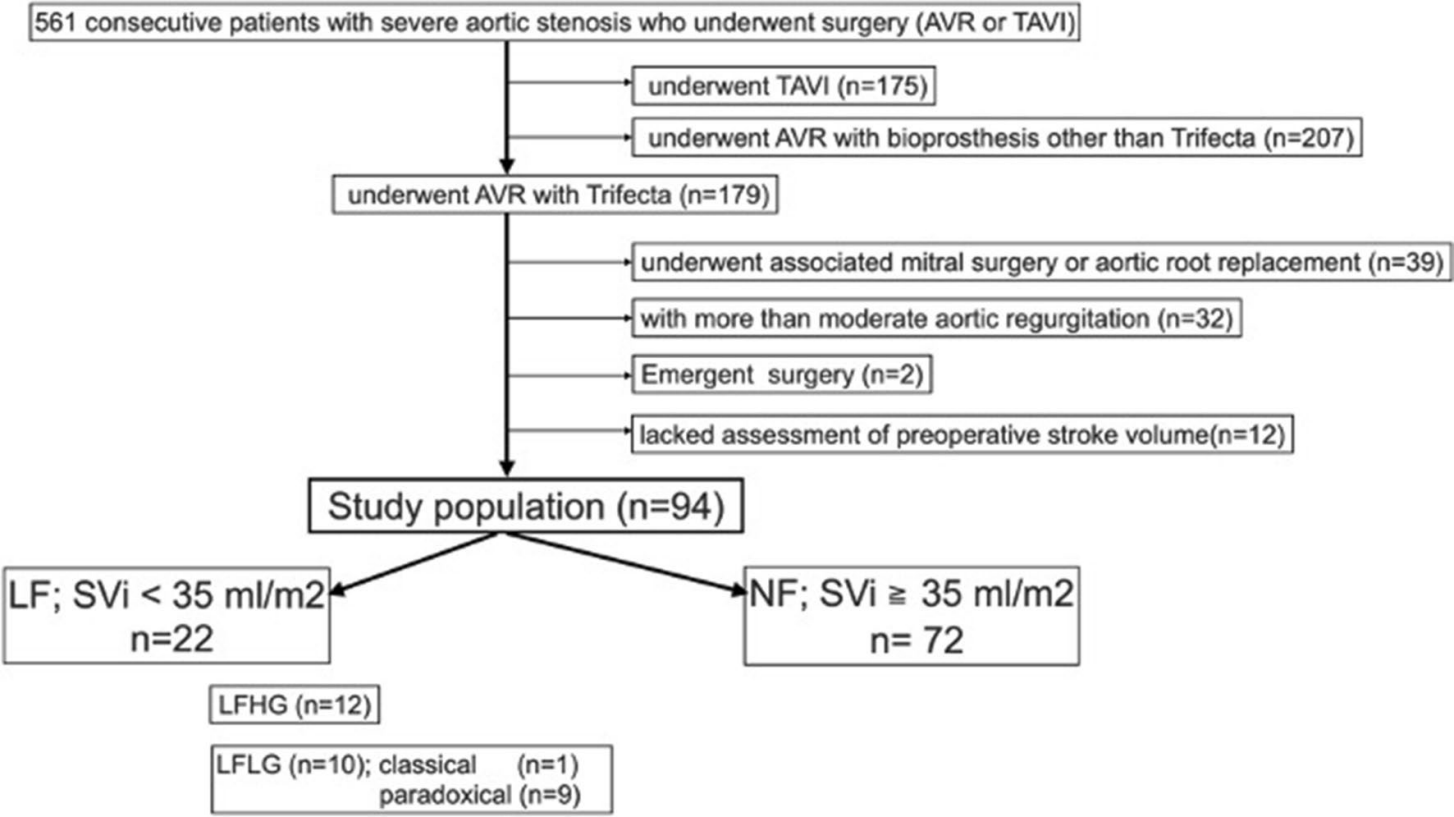
Patients flow chart

This was a single-center retrospective study. From September 2012 to September 2020, 561 consecutive patients with severe AS who underwent surgical treatment (AVR or TAVI) at Kurume University Hospital were analyzed (Fig. 1). Among them, the following patients were excluded from the analysis: 175 patients who underwent TAVI (TAVI started since 2014 in our hospital); 207 patients who underwent AVR other than Trifecta (valve selection was depend on surgeon’s preference); 39 patients who underwent associated mitral surgery or aortic root replacement; 32 patients with more than moderate aortic regurgitation; two patients underwent emergent surgery; 12 patients who lacked assessment of stroke volume before surgery. Finally, 94 patients were included for analysis. The study population was divided into two groups according to the stroke volume index (SVI): the low-flow (LF) group (SVI < 35 ml/m^2^, *n* = 22) and the normal-flow (NF) group (SVI ≥ 35 ml/m^2^, *n* = 72). LF group included ten patients with low-flow (SVI < 35 ml/m^2^) and low gradient (mean PG < 35 mmHg) (LFLG). LFLG patients included nine paradoxical (EF > 50%) LFLG and 1 classical LFLG (EF ≥ 50%) patients. The patients’ clinical characteristics are shown in Table 1. The Ethics Committee of Kurume University approved this study (20,037) on 28 May 2020.

**Table 1:** Preoperative patient characteristics

All (*n* = 94)	LF (*n* = 22)	NF (*n* = 72)	*P* value
Age (years), mean ± SD	81.5 ± 4.6	80.5 ± 3.9	0.17
80 years≧, *n* (%)	17 (77.2)	48 (66.7)	0.35
Male gender, *n* (%)	8 (36.4)	22 (30.6)	0.61
Body surface area (m^2^), mean ± SD	1.46 ± 0.14	1.45 ± 0.16	0.71
Hypertension, *n* (%)	19 (86.4)	65 (90.3)	0.6
Hyperlipidemia, *n* (%)	15 (68.2)	41 (56.9)	0.35
Diabetes mellitus, *n* (%)	9 (40.9)	21 (29.2)	0.3
Smoking, *n* (%)	5 (22.7)	7 (9.7)	0.11
COPD, *n* (%)	5 (22.7)	17 (23.6)	0.93
Hemodialysis, *n* (%)	0 (0)	4 (5.4)	0.14
History of cardiac surgery, *n* (%)	0 (0)	0 (0)	-
atrial fibrillation, *n* (%)	4 (17.4)	3 (4.1)	0.048
ACEI or ARB use, *n* (%)	6 (27.2)	26 (36.1)	0.41
β blocker use, *n* (%)	14 (63.6)	44 (61.1)	0.9
calcium channel blocker, *n* (%)	3 (13.6)	19 (26.4)	0.19
New York Heart Association Class	2.0 ± 0.6	1.9 ± 0.6	0.49
SBP (mmHg), mean ± SD	120 ± 25	138 ± 21	< 0.01
DBP (mmHg), mean ± SD	67 ± 15	72 ± 12	0.11
Heart rate (bpm), mean ± SD	71.0 ± 10.1	66.4 ± 10.8	0.08
eGFR (ml/min/1.73 m^2^), mean ± SD	57.8 ± 20.2	58.1 ± 24.2	0.96
Hemoglobin (mg/dl), mean ± SD	12.5 ± 1.8	11.9 ± 1.6	0.12
Bicuspid valve, n (%)	2 (9.1)	9 (12.5)	0.66

*COPD* chronic obstructive pulmonary disease, *ARB* angiotensin II receptor blocker, *ACEI* angiotensin-converting enzyme inhibitor, *SBP* Systolic blood pressure, *DBP* Diastolic blood pressure, *eGFR* Glomerular filtration rate, *SD* standard deviation

### Echocardiography

Echocardiographic data were obtained with commercially available ultrasound systems. All patients underwent a comprehensive examination, including M-mode and two-dimensional echocardiography and Doppler examinations. All tests were conducted by experienced sonographers. The aortic valve area was calculated using the continuity equation. The left ventricular ejection fraction (LVEF) was calculated using the Teichholz method and LV mass was calculated according to the Devereux formula [11]. The SVI was estimated by multiplying the LV outflow tract area by the LV outflow tract velocity–time integral on pulsed-wave Doppler recordings and was then indexed to the body surface area. The patients’ preoperative echocardiographic data are shown in Table 2. In 58 patients (59%, 12 in the LF group and 46 in the NF group) who obtained echocardiographic results 1 year after AVR, a comparative study was performed before and 1 year after surgery. Categorization of PPM was based on the indexed effective orifice area (EOAi), with severe PPM defined as an EOAi < 0.65 cm^2^/m^2^ and moderate PPM as an EOAi ≥ 0.65 and ≤ 0.85 cm^2^/m^2^. Comparison of echocardiographic data between preoperatively and postoperatively at 1 year is shown in Table 3.

**Table 2:** Preoperative echocardiographic analysis

All (*n* = 94)	LF (*n* = 22)	NF (*n* = 72)	*P* value
EF (%), mean ± SD	62.9 ± 13.5	69.4 ± 11.3	0.02
LVDd, mean ± SD	41.8 ± 5.1	44.2 ± 5.3	0.04
LVDs (mm), mean ± SD	27.7 ± 6.7	26.8 ± 5.8	0.57
IVST (mm), mean ± SD	12.2 ± 2.8	11.8 ± 1.8	0.87
PWT (mm), mean ± SD	11.4 ± 2.4	11.6 ± 1.5	0.32
PV (m/s), mean ± SD	4.0 ± 1.1	4.5 ± 0.9	< 0.01
max PG (mmHg), mean ± SD	67.6 ± 35.7	89.4 ± 32.9	< 0.01
mean PG (mmHg), mean ± SD	38.6 ± 23.3	51.2 ± 19.5	< 0.01
AVA (cm^2^), mean ± SD	0.60 ± 0.2	0.66 ± 0.2	0.26
AR; none to trivial, *n* (%)	13 (59)	36 (50)	0.61
AR; mild, *n* (%)	9 (41)	36 (50)	0.61
AR; more than moderate, *n* (%)	0	0	–
LVMI (g/m^2^), mean ± SD	142.3 ± 47.6	153.3 ± 40.6	0.19
SVI (mL/m^2^), mean ± SD	29.1 ± 4.7	49.3 ± 10.1	< 0.01

*EF* Ejection fraction, *LVDd* Left ventricular end-diastolic diameter, *LVDs* Left ventricular end-systolic diameter, *IVST* interventricular septal thickness, *PWT* posterior left ventricular wall thickness, *PV* peak velocity, *PG* pressure gradient, *AVA* aortic valve area, *AR* aortic regurgitation, *LVMI* left ventricular mass index, *SVI* stroke volume index, *SD* standard deviation

**Table 3:** Preoperative versus 1-year follow-up echocardiographic analysis

	LF (*n* = 12)			NF (*n* = 46)		
	Pre	1 year	*P* value	Pre	1 year	*P* value
EF (%),mean ± SD	63.5 ± 12.0	67.2 ± 8.7	0.54	70.1 ± 10.3	69.9 ± 6.8	0.36
LVDd (mm), mean ± SD	41.3 ± 4.8	39.2 ± 4.5	0.66	44.7 ± 5.0	40.5 ± 5.4	< 0.01
LVDs (mm), mean ± SD	27.2 ± 5.5	25.3 ± 4.7	0.37	26.8 ± 5.2	24.6 ± 4.3	< 0.01
IVST (mm), mean ± SD	10.9 ± 2.0	9.9 ± 1.8	0.31	11.8 ± 1.9	10.7 ± 1.5	< 0.01
PWT (mm), mean ± SD	10.7 ± 1.7	9.8 ± 1.5	0.32	11.7 ± 1.5	10.5 ± 1.4	< 0.01
PV (m/s), mean ± SD	3.9 ± 0.9	2.0 ± 0.3	< 0.01	4.6 ± 0.9	2.2 ± 0.4	< 0.01
max PG (mmHg), mean ± SD	62.6 ± 31.0	17.1 ± 4.0	< 0.01	88.7 ± 33.8	21.0 ± 6.1	< 0.01
mean PG (mmHg), mean ± SD	35.2 ± 22.0	8.3 ± 1.7	< 0.01	51.3 ± 20.1	10.6 ± 3.0	< 0.01
AVA (cm^2^), mean ± SD	0.66 ± 0.21	1.57 ± 0.3	< 0.01	0.67 ± 0.15	1.42 ± 0.24	< 0.01
LVMI (g/m^2^), mean ± SD	122.7 ± 41.0	98.3 ± 23.9	0.11	156.7 ± 40.2	113.2 ± 28.5	< 0.01
PPM						
Moderate	–	0		–	6 (13.3)	0.09
Severe	–	0		–	0	-

*EF* Ejection fraction, *LVDd* Left ventricular end-diastolic diameter, *LVDs* Left ventricular end-systolic diameter, *IVST* interventricular septal thickness, *PWT* posterior left ventricular wall thickness, *PV* peak velocity, *PG* pressure gradient, *AVA* aortic valve area, *LVMI* left ventricular mass index, *PPM* Patient-prosthesis mismatch, *SD* standard deviation

### Aortic valve replacement with Trifecta bioprosthesis

AVR was performed by full sternotomy using standard cardiopulmonary bypass. Myocardial protection was achieved with cold crystalloid antegrade cardioplegia. The size of the Trifecta bioprosthesis was determined by the surgeon and guided by the manufacturer-supplied replica sizer. The Trifecta bioprosthesis was sewn in a supra-annular position using a non-everting mattress suture. The operative information, including concomitant procedures, is shown in Table 4.

**Table 4:** Intraoperative and postoperative data

All (*n* = 94)	LF (*n* = 22)	NF (*n* = 72)	*P* value
Operation time (min), mean ± SD	336 ± 86	300 ± 116	0.02
CPB time (min), mean ± SD	167 ± 62	144 ± 52	0.11
Cross-clamp time (min), mean ± SD	115 ± 38	102 ± 33	0.09
Concomitant procedure			
CABG, *n* (%)	6 (27.3)	25 (34.7)	0.51
Tricuspid valve, *n* (%)	2 (9.1)	1 (1.4)	0.11
Aorta, *n* (%)	2 (9.1)	4 (5.6)	0.33
Arrhythmia, *n* (%)	3 (13.6)	4 (5.6)	0.24
Bioprostheses valve size, *n* (%)			
19 mm	10 (45.5)	40 (55.6)	0.59
21 mm	10 (45.5)	24 (33.3)	
23 mm	2 (9.1)	8 (11.1)	
Length of ICU stay (days), mean ± SD	3.1 ± 0.8	2.8 ± 1.2	0.07
Length of hospital stay (days), mean ± SD	27.4 ± 17.8	22.3 ± 12.0	0.1
Postoperative ventilation time (hour), mean ± SD	20.9 ± 17.6	24.1 ± 38.1	0.97
Postoperative ventilation time > 72hour, *n* (%)	2 (8.3)	0 (0)	0.12
Complication, n (%)			
Stroke, *n* (%)	1 (4.5)	3 (4.2)	0.93
Atrial fibrillation, *n* (%)	9 (40.9)	18 (25.0)	0.16
Surgical site infection	0 (0)	4 (5.6)	0.85
Hospital mortality	0 (0)	0 (0)	–

*CPB* Cardiopulumonary bypass, *CABG* coronary artery bypass grafting, *ICU* intensive care unit, *SD* standard deviation

### Mortality and clinical follow-up

Hospital mortality was defined as death within 30 days after AVR. Overall mortality was defined as the combination of hospital mortality and late mortality. Adverse cardiovascular events defined according to the VARC-2 (Valve Academic Research Consurtium-2) consensus [12] were retrospectively extracted from patients’ electronic health records. The clinical follow-up rate was 100%, with a mean follow-up of 1041 days in the LF group and 1037 days in the NF group.

### Statistical analysis

Continuous variables are expressed as mean ± standard deviation. The Fisher’s exact test and the *χ*^2^ test with Yates correction were used to compare categorical variables. The Wilcoxon test was used as appropriate for continuous variables. Long-term survival was assessed using the Kaplan–Meier survival curve, and differences were assessed with the log-rank test. Statistical analyses were performed using JMP 13 software (SAS Institute Japan Ltd., Tokyo, Japan). A value of *p* < 0.05 was considered statistically significant.

## Results

### Baseline characteristics

A total of 23% of our cohort were in the LF group. There were no significant differences in age, sex, body surface area, comorbidities, and New York Heart Association functional class between the two groups. The mean age was older than 80 years in both groups. Mean systolic blood pressure in the NF group was significantly higher than that in the LF group (LF:NF = 120:137 mmHg, *p* < 0.01), despite no significant difference in preoperative medical therapy between the groups. The number of patients with atrial fibrillation was significantly higher in the LF group than in the NF group (*p* = 0.048). No significant differences in diastolic blood pressure, heart rate, preoperative renal function, and anemia were found between the two groups (Table 1).

### Preoperative echocardiography

There were no significant differences in left ventricular end-systolic diameter (LVDs), interventricular septal thickness (IVS), posterior left ventricular wall thickness (PWT), and the left ventricular mass index (LVMI) between the two groups. The LVEF and left ventricular end-diastolic diameter (LVDd) were significantly lower in the LF group than in the NF group. The peak pressure gradient, mean gradient, and peak velocity across the aortic valve were lower in the LF group than in the NF group. However, the area of the aortic valve was similar in both groups. No patients had more than moderate AR. The SVI was significantly smaller in the LF group than in the NF group (LF:NF = 29.1:49.3 ml/m^2^, *p* < 0.0001) (Table 2).

### Operative outcomes

The sizes of the Trifecta bioprosthetic valve used were 19, 21, and 23 mm in 10, 10, and 2 patients in the LF group, respectively, and 19, 21, and 23 mm in 40, 24, and eight patients in the NF group, respectively. The Trifecta bioprosthesis size was similar in both groups and the proportion of patients with a small root who had the 19-mm bioprosthesis implanted was approximately 50% in both groups. Concomitant procedures were also similar in both groups. The operative time was significantly longer in the LF group than in the NF group; however, cardiopulmonary bypass time and aortic cross-clamp time were not significantly different between the two groups. There was no hospital mortality in both groups. The postoperative course and the mobility rate postoperatively were also not different between the groups (Table 4).

### Echocardiographic outcomes at 1 year

No patients had severe PPM in either group. The rate of moderate PPM was 13% in the NF group and no patients had PPM in the LF group. The LVEF did not change significantly at 1 year after AVR in both groups. LVDd, LVDs, IVST, PWT, and LVMI became significantly smaller at 1 year after AVR in the NF group, but these did not significantly change in the LF group. Peak velocity, and peak and mean pressure gradients across the aortic valve were improved at 1 year after AVR in both groups. (Table 4).

### Mid-term clinical outcome

**Fig. 2:**
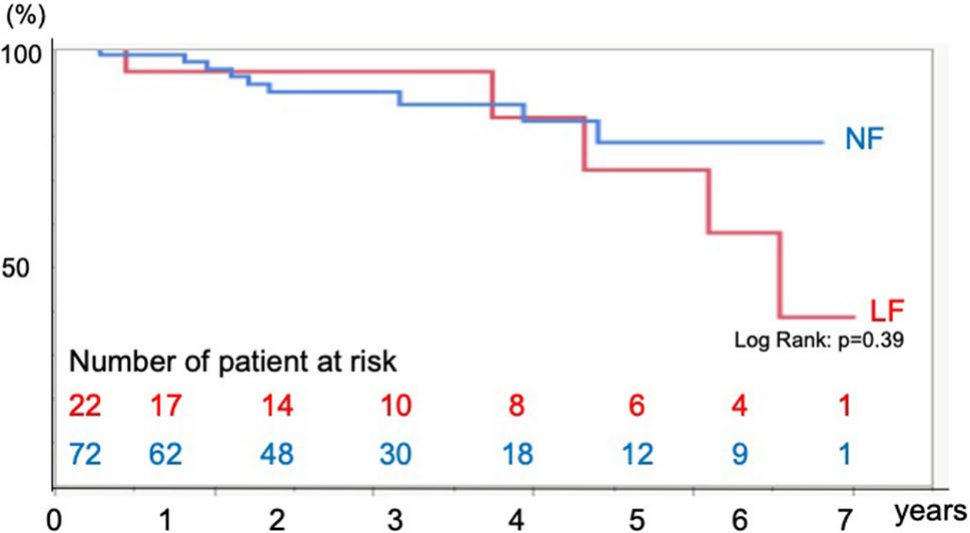
Kaplan–Meier curve of the survival rate after AVR in the LF and NF groups

**Fig. 3:**
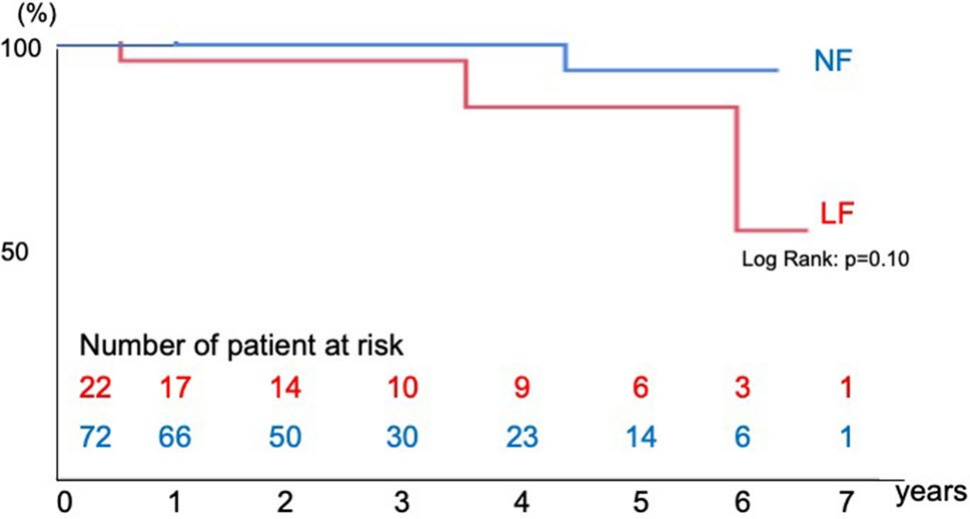
Kaplan–Meier curve of the cardiovascular event-free rate after AVR in the LF and NF groups

**Fig. 4:**
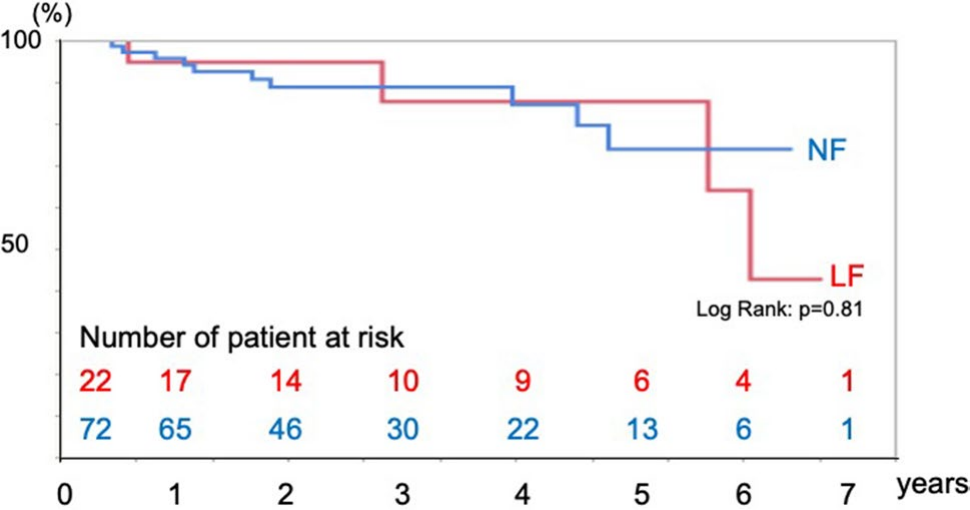
Kaplan–Meier curve of the cardiovascular death-free rate after AVR in the LF and NF groups

Fifteen death occurred in the follow-up period. Ten patients died in the NF group and five patients died in the LF group in the follow-up period. There were no significant differences in the overall survival, cardiovascular death-free, and cardiovascular event-free rates between the two groups. Kaplan–Meier analysis showed no significant differences in the survival rate (log-rank test, *p* = 0.39) (Fig. 2), cardiovascular death-free rate (log-rank test, *p* = 0.10) (Fig. 3), and cardiovascular event-free rate (log-rank test, *p* = 0.81) (Fig. 4) between the two groups. The 5-year survival rate, cardiovascular death-free rate, and cardiovascular event-free rate were 72.2%, 90.0%, and 90.0% in the LF group, respectively, and 78.5%, 94.4%, and 86.3% in the NF group, respectively. Structural valve deterioration (SVD) occurred in one patient in the NF group at 3 years after AVR, and she underwent re-AVR using another bioprosthesis.

## Discussion

Severe AS with LV dysfunction has a poor prognosis according to the European Society of Cardiology/European Association for Cardio-Thoracic Surgery guideline [13]. LV function is an important factor that affects the outcome of patients with severe AS who undergo AVR or TAVI. However, patients with severe AS often have LV hypertrophy, and evaluating their LV function with the LVEF alone is difficult. Low flow across the aortic valve is a multifactorial phenomenon that includes impaired myocardial contractility, restrictive physiological features, and afterload mismatch with high arterial impedance. The SVI has recently been considered useful for evaluating LV function [14]. Many reports have also shown that a low SVI is associated with the prognosis of severe AS after surgical treatment (AVR or TAVI). The SVI is a recognized feature that aids workup and management of AS [1–6]. In the present study, almost a quarter of the patients with severe AS had a low SVI. This percentage is slightly lower than previously reported data in which the incidence of low flow ranged from 30 to 55% [6]. The patients in the present study were divided into the LF and NF groups according to the SVI. There were no significant differences in the preoperative characteristics between the LF and NF groups with the exception of the systolic blood pressure and the rate of atrial fibrillation. A preoperative echocardiogram showed no significant difference in the LVMI between the two groups. The preoperative LVEF and the preoperative peak and mean pressure gradients across the aortic valve were lower in the LF group than in the NF group. These preoperative echocardiographic results suggest that LV function was worse in the LF group than in the NF group. These differences between the LF and NF groups may reflect differences in the patients’ backgrounds. Different prognoses are expected because patients with low LV function have more advanced disease. We initially considered that the LF group might have worse outcomes than the NF group both operatively and in terms of the mid- to long-term prognosis. However, the results of AVR with the Trifecta bioprosthesis in this study showed no significant difference in the operative or mid-term outcome between the two groups. This finding may indicate that the Trifecta bioprosthesis is effective for patients with low-flow AS.

Some studies have shown that operative mortality or long-term survival is worse in patients with low-flow AS than in those with normal-flow AS who undergo AVR or TAVI. Lopez-Marco et al. [1] studied 198 patients with isolated AVR whose mean age was about 70 years. These authors reported that AVR in patients with low-flow AS was associated with similar surgical mortality but higher mid-term mortality than in patients with normal-flow AS. Fan et al. [2] studied 863 patients with a mean age in the fifth decade of life. These authors reported that hospital mortality was higher and the 5-year survival rate was lower in the LF group than in the NF group. Fukui et al. [3] studied 179 patients with AVR with a mean age in the seventh decade of life. In their study, the patients only had normal LV function, and most had undergone implantation of a 19-mm valve. Hospital mortality was significantly higher and 5-year overall survival was lower in the LF group than in the NF group [3], similar to other reports. Kataoka et al. [4] studied 723 patients with a mean age of 85 years who had undergone TAVI. These authors found that the mid-term rate of death of all causes and cardiovascular causes was higher in the LF group than in the NF group. Le Ven et al. [5] studied 639 patients with a mean age of 80 years who had undergone TAVI and found that the 2-year all-cause mortality rate was worse in the LF group than in the NF group. Mangner et al. [6] studied 1600 patients who had undergone TAVI and found that the 3-year all-cause mortality rate after TAVI was worse in the LF group than in the NF group. These previous reports differ from our study regarding certain patients’ characteristics, such as age. Moreover, most of these previous reports did not show the incidence of PPM after surgery (AVR or TAVI). Only Lopez-Marco et al. [1] reported a PPM rate of 2% in their NF group and 6% in their LF group.

The incidence of PPM is an important risk factor for long-term survival after AVR. PPM leads to poor hemodynamic valve performance (i.e., elevated transvalvular pressure gradient) despite a fully functioning prosthesis and is associated with poor clinical outcomes, including long-term survival, a low rate of freedom from heart failure, and poor LV mass regression [15]. The small annulus and aortic root in many older patients allow implantation of a small prosthesis, which is often associated with a high trans-prosthetic gradient, small EOA, and high incidence of PPM. A previous study showed that PPM was associated with increased operative mortality after AVR, particularly when associated with LV dysfunction [8]. In the present study, most patients had a small body mass because the mean age was older than 80 years and the proportion of women was high. Therefore, more than half of the patients underwent implantation with a small-diameter Trifecta bioprosthesis (19 or 21 mm). However, an echocardiogram at 1 year after AVR showed no severe PPM in either group, and moderate PPM occurred in six patients only in the NF group. Because of the issue of PPM in AVR, the valve design has been constantly evolving in an effort to improve the EOA. In a previous study, severe PPM (EOAi of < 0.60 cm^2^/m^2^) was detected in 3% of patients with the Trifecta bioprosthesis [16]. The favorable hemodynamics of the Trifecta bioprosthesis may decrease the occurrence of PPM. Decreased occurrence of PPM may have contributed to regression of the LVMI at 1 year after AVR in the LF and NF groups in our study. Selection of an appropriate bioprosthesis to avoid PPM in our patient population may have led to good mid-term results.

Some reports have shown that the Trifecta bioprosthesis is associated with a higher occurrence of AVR for SVD [17, 18]. The rate of SVD was lower in the present study than in other reports [17, 18], but surgeons need to be aware of the occurrence of SVD when AVR is performed with the Trifecta bioprosthesis. Therefore, we attempted to use the Trifecta bioprosthesis more actively in patients aged > 80 years. The LVMI is reduced and a favorable prognosis can be expected by avoiding PPM until occurrence of SVD. We may perform TAVI with a surgical aortic valve in older patients when SVD occurs. However, particularly in the small prosthesis size and also in degenerated bioprosthesis with external leaflet, mounting a TAVI in surgical aortic valve procedure is associated with a higher risk of coronary obstruction. The advantages and disadvantages of the Trifecta bioprosthesis should be carefully considered.

This study has several limitations. First, it was a retrospective single-center study with a small sample size. As a result, a test for non-inferiority between the groups was not performed. The possibility of a type 2 error should be considered in this study. Second, the LF group was not compared with patients who underwent AVR using other bioprosthetic valves. Third, measurements of the LV outflow tract [10], mitral regurgitation, atrial fibrillation, and image quality have been shown to affect calculation of the SVI. Finally, patients with coronary artery disease undergoing AVR + CABG were included in the study. Remodeling as a consequence of revascularization may have occurred in these patients. However, we believe that it is clinically meaningful to be able to present favorable retrospective data from a single center.

In conclusion, no patients had severe PPM, and LV hypertrophy improved at 1 year after AVR with the Trifecta bioprosthesis in the LF and NF groups. As a result, there were no significant differences in surgical early or mid-term outcomes between the groups. The favorable hemodynamic performance of the Trifecta bioprosthesis may have resulted in the same operative outcomes in the LF and NF groups. AVR with the Trifecta bioprosthesis might be considered for avoidance of PPM, particularly in patients with AS who have LV dysfunction. Further prospective studies are needed to clarify selection of the optimal bioprosthesis in patients with AS.
